# Supplementary material for: Photoinduced bond oscillations in ironpentacarbonyl give delayed synchronous bursts of carbonmonoxide release
Source: Nat Commun. 2022 Mar 14;13:1337. doi: 10.1038/s41467-022-28997-z (PMC8921231; doi:10.1038/s41467-022-28997-z)
Supplement: Supplementary file 1 — Supplementary Information [file 41467_2022_28997_MOESM1_ESM.pdf]

Supplementary Information for Photoinduced bond oscillations in ironpentacarbonyl give delayed, synchronous bursts of carbonmonoxide release

Ambar Banerjee,[1,*] Michael R. Coates,[1] Markus Kowalewski,[1] Hampus Wikmark,[2] Raphael M. Jay,[2] Philippe Wernet,[2] and Michael Odelius[1,†]

[1] *Department of Physics, Stockholm University, AlbaNova University Center, SE-106 91 Stockholm Sweden*
[2] *Department of Physics and Astronomy, Uppsala University, Box 516, SE-751 20 Uppsala, Sweden*

[*] ambarpchem@gmail.com
[†] odelius@fysik.su.se

SUPPLEMENTARY NOTES 1

Correctness and Limitations of TDDFT in Comparison with NEVPT2: TDDFT and NEVPT2 are two completely different theories and hence the difference between them is very well expected, and that too for a transition metal system. The accuracy of TDDFT and NEVPT2 spectra when compared to the experimental spectra are similar but in opposite direction, i.e., red shifted in TDDFT and blue shifted in NEVPT2. This difference in vertical excitation energies is due to approximations in TDDFT, the use of a rather limited active space, and the difference in accounting for the correlation energy of the ground state with respect to the excited state, which results in error in estimation of the vertical excitation energy. This is depicted by the fact that MRCI(8e,16o) excitation energies as reproduced by Daniel and coworkers [14] are much closer to the TDDFT excitation energies than NEVPT2/CASPT2(10e,10o) energies. We are however not interested in the energy with respect to the ground state during the dynamics itself. Rather the excited state dynamics is governed by the relative energetics between the excited states and the shapes of these potentials. The relative energy of excited states, i.e., for example between the bright $^{1}A_2$" (MLCT) and dark $^{1}E'$ (MC) states are consistent for TDDFT and NEVPT2, and those are the states largely driving the dynamics. Though there is reordering of the states among themselves also, if we look carefully at Supplementary Table 1, the ordering is among those states which are quasi-degenerate by less that 0.5 eV, and that is most likely the result of the difference in treatment of correlation energy in TDDFT vs NEVPT2 level of theory. We would also like to mention here that the variety of functionals explored for TDDFT is rather limited in the present study, and the aforementioned trend is limited to the three functional mentioned in Supplementary Table 1. This may change for different functionals. At present point we are limited to TDDFT, but based on the present results, we are evaluating the possibility to further investigate the grid-based quantum dynamics in reduced dimension on a higher level of theory (e.g., NEVPT2). It is noteworthy to mention here that when we began the excited state MD simulation, we did so at the CASSCF level of theory (i.e., from the S_8 which is $^{1}A_2$" (MLCT) in CASSCF) state and that resulted in dissociation of double CO dissociation, with direct formation of $Fe(CO)_3$. This is in direct contradiction to what is experimentally observed, indicating that dynamically correlation is important. Thus, TDDFT was the method of choice for our study.

$Fe(CO)_5$ is a closed shell system with a singlet ground state, and hence conceptually TDDFT is applicable to it in dealing with the excited states. However, $Fe(CO)_4$ has a triplet ground state and thus with such systems the singlet ~~ground~~ S_0 state often has a multideterminantal wavefunction, (for example O_2). We are studying the dynamics from $Fe(CO)_5$ to $Fe(CO)_4$ and hence operating in a fuzzy zone when it comes to the extent the dynamics is correct and is not. We started the dynamics with CASSCF(10e,10o) and found it always gave simultaneous double dissociation which is contradicted by experiment and hence was not abandoned. The reason for the failure of CASSCF dynamics is the importance of dynamical correlation in the description of excited states of $Fe(CO)_5$. This is already discussed in details in the main manuscript and shown by the flipping of roots as one goes from CASSCF to NEVPT2/CASPT2, as depicted in Supplementary Table 1. Hence TDDFT is the method of choice and it agrees with UV-vis qualitatively and resembles NEVPT2/CASPT2 and MRCI (from Ref. [14]) excited state manifold, with obvious disagreements. The limitations of TDDFT are broadly two-fold; one, when the ground state has multideterminant wavefunction and secondly in dealing with double excitations. $Fe(CO)_4$ suffers from the first limitation and hence excluded from our study. When it comes to the second reason the excited states of $Fe(CO)_5$ are single excitation ones as confirmed from CASSCF computation, and hence TDDFT is applicable. Moreover TDDFT has been used earlier in dealing with the dynamics of $Cr(CO)_6$ (a close enough system) by Barbatti and co-workers [31].

Additionally, we have taken one of the trajectories and have conducted NEVPT2 computations on configurations sampled every 5 fs. Following the computation, the TDDFT and NEVPT2 energies were plotted as a function of Fe-C distance, as shown in Supplementary Figure 4 (left panel). The similarities from the two theories include the separation between the MLCT states and the MC states which increases as the Fe-C bond dissociates. This also confirms the picture that dissociation happens in the MC states. Obviously, the slope for NEVPT2 as revealed is a bit steeper and hence the dynamics at NEVPT2 level of theory could give a faster dissociation, but one must keep in mind that the dynamics was done at TDDFT and hence were based on TDDFT gradient, the pathway will be somewhat different with NEVPT2 gradients, which are currently unavailable. Now the most important disagreement, that we clearly see from Supplementary Figure 4, is that as Fe-C distance increases the disagreement between the TDDFT and NEVPT2 increase when it comes to the MLCT states. This is primarily due to fact that TDDFT fails for $Fe(CO)_4$ as discussed earlier. However, as the disagreement is mainly more pronounced in the MLCT state whereas most of the dynamics happens on the MC states, which we expect are theoretically sound except at very large distance where errors due to a multi-reference wavefunction takes hold.

State	TDDFT/ CAM-B3LYP	TDDFT/ wB97XD	TDDFT/ B3LYP	NEVPT2 (10e,10o)	CASPT2 (10e,10o)	CASPT2 (10e,10o) (no IPEA) Ref.[33]	CASSCF (10e,10o)	MRCI (8e,16o) Ref.[14]
$^1A_1''$(MLCT)	3.89 (0.000)	3.96(0.000)	3.74(0.000)	4.98 (0.000)	4.88 (0.000)	4.40	5.7	4.12
$^1E'$(MC)	3.97 (0.000)	3.99(0.000)	3.98(0.000)	4.65 (0.001)	4.48 (0.001)	4.21	5.0	3.55
$^1E''$(MLCT)	4.17 (0.000)	4.25 (0.000)	3.99 (0.000)	5.22 (0.000)	5.12 (0.000)	4.59	6.48	4.55
$^1A_2''$(MLCT)	4.40 (0.067)	4.51 (0.070)	4.09 (0.052)	5.17 (0.141)	5.11 (0.150)	4.53	6.63	4.59
$^1A_1'$(MLCT)	4.53 (0.000)	4.62 (0.000)	4.31 (0.000)	—	—	—	—	—
$^1E'$(MLCT)	4.66 (0.019)	4.75 (0.022)	4.38 (0.007)	6.01 (0.130)	6.33 (0.091)	5.25	7.3	4.87

Supplementary Table 1. **Vertical excitation energies (eV) for the CASPT2 optimized geometry** Vertical excitation energies (eV) at the TDDFT, NEVPT2, CASPT2 and CASSCF levels of theory of states involved in the excited state molecular dynamics simulations are mentioned in this table. Computational details are stated in Methods Section in the main text. The oscillator strengths are provided in parenthesis alongside the energies. Please refer to Supplementary Figure 1 below for the character of the states in terms of orbital transition involved in the different excited states.

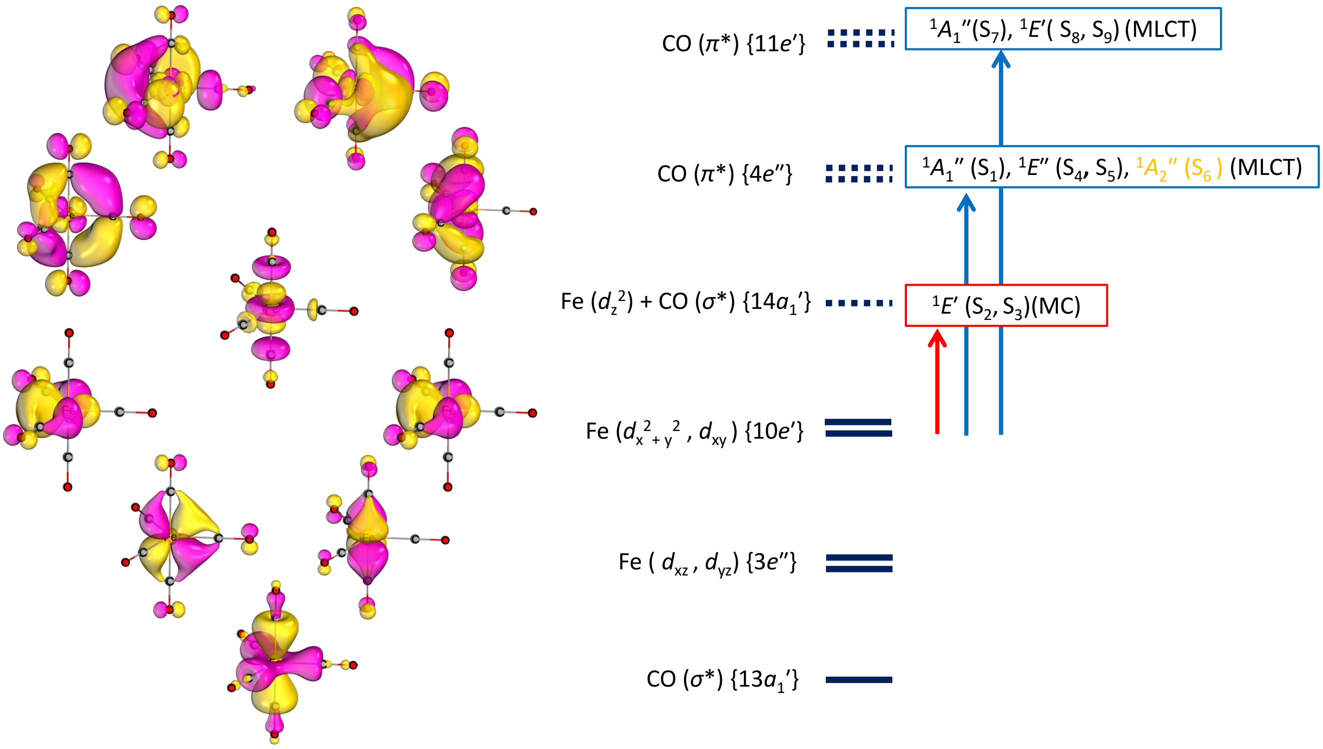

Supplementary Figure 1. **Molecular orbitals and excited state character for the Frank-Condon transitions in $Fe(CO)_5$.** In the left hand side of the figure we show the MOs from the CASSCF(10e,10o) computation. These also represents the molecular orbitals which are involved in the major contribution to the exited manifold reproduced by TDDFT. The right hand side of the figure shows the character of the states as excitation from one occupied molecular orbital (represented by solid lines) to an unoccupied molecular orbital (represented by dashed line). Kindly refer to Supplementary Table 1 for the energies of the states and comparison between different theories.

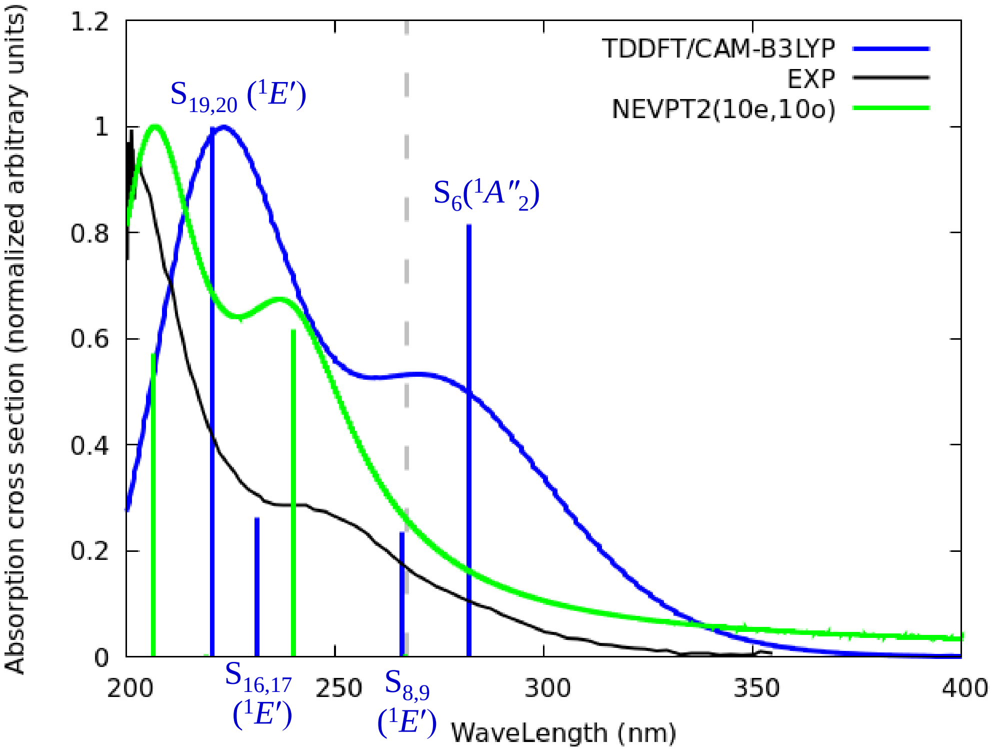

Supplementary Figure 2. **UV-vis of gas phase Fe(CO)$_5$.** We show here computed UV-vis absorption spectra of gas phase Fe(CO)$_5$ at TDDFT/CAM-B3LYP (blue) and NEVPT2 (green) level of theory, obtained from the transitions in Supplementary Table 1 by convolution with a 0.8 eV full-width half-maximum Gaussian function. (They are calculated at the equilibrium geometry and the TDDFT curve in this graph is reproduced from Figure 1 in the main text.) The convoluted spectra are compared to the experimental Fe(CO)$_5$ UV-vis spectra in gas phase (black) as given in the Ref. [32]. The gray dashed line depicts the wave length corresponding to a 267 nm excitation, which is near the position of the peak at 250 nm in the experimental spectrum. Bright discrete states in the TDDFT calculation are included and denoted by state number and symmetry labels. Computational details are stated in Methods Section in the main text.

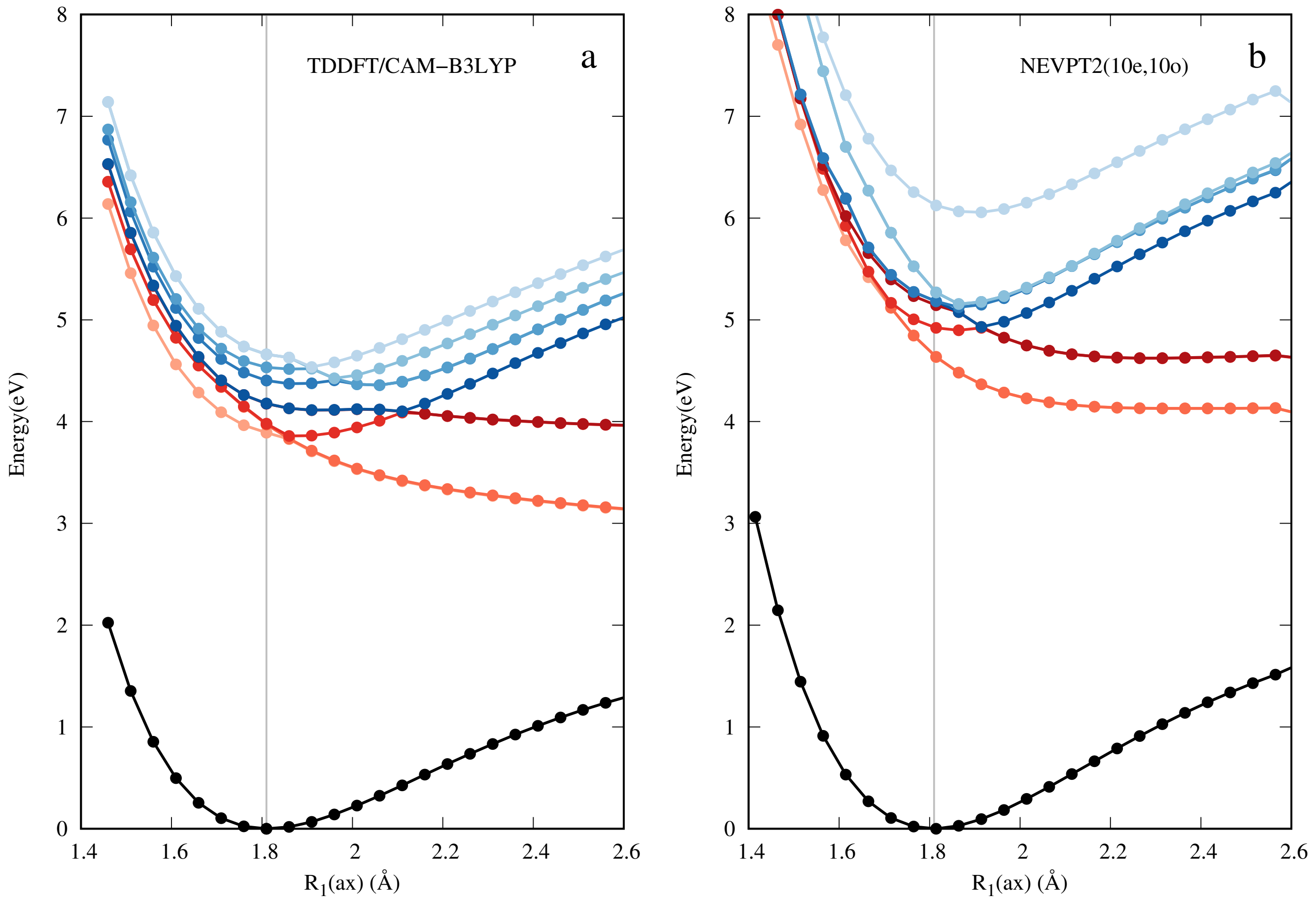

Supplementary Figure 3. **Adiabatic potential energy surfaces for rigid scan corresponding to the dissociation of axial Fe-CO bond** a) TDDFT/CAM-B3LYP adiabatic potential energy surfaces of the 10 lowest lying electronic singlet states plotted against varying an axial Fe-C distance starting from the equilibrium D_{3h} geometry (see Methods Section in main text), and distorted within C_{3v} symmetry in a rigid scan displacing a CO ligand. These one dimensional cuts correspond to the two dimensional cuts presented in Supplementary Figure 10. b) The corresponding NEVPT2 data. The ground state potential is black and the dissociative states are presented in various shades of red, whereas the non-dissociative state are shown in different shades of blue. The vertical gray line represents the equilibrium distance.

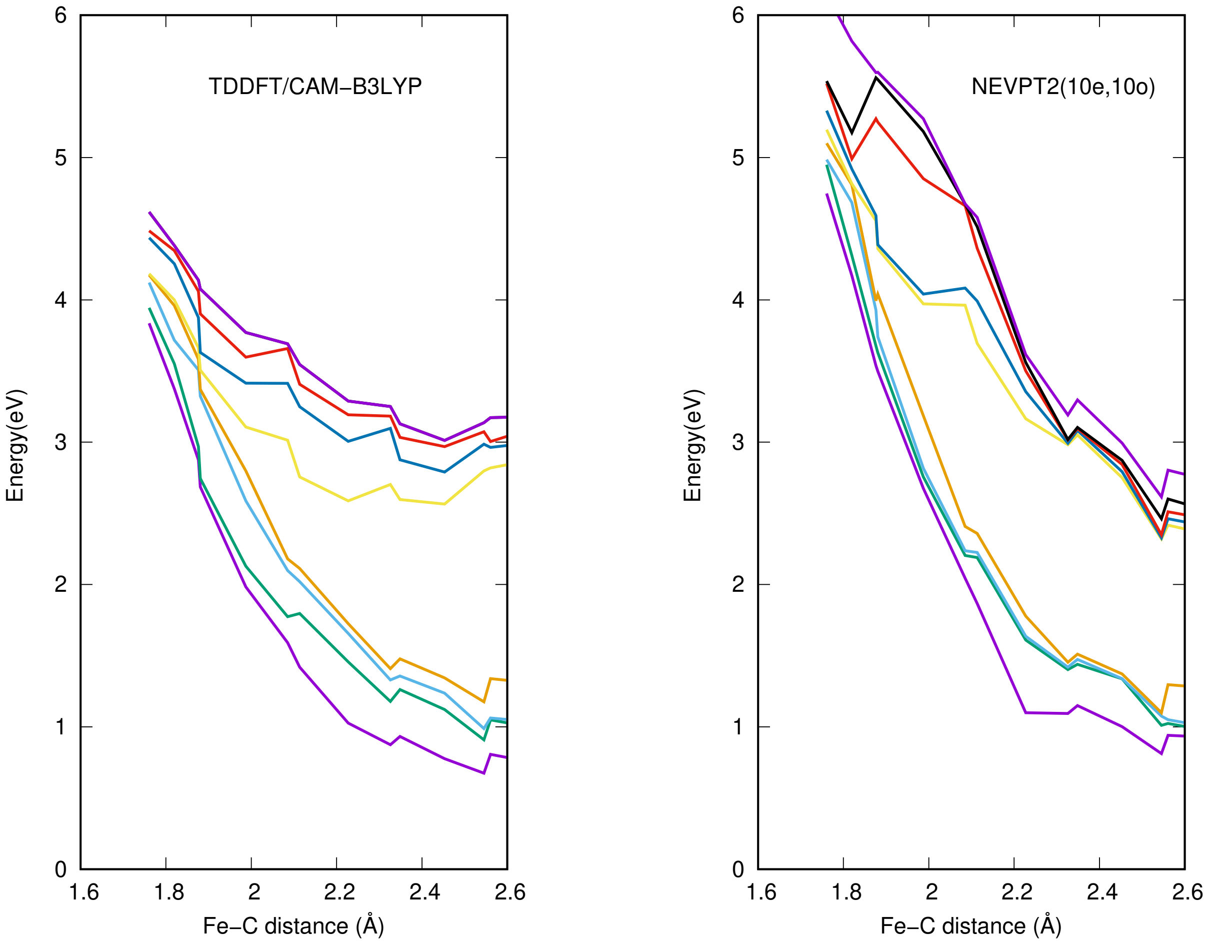

Supplementary Figure 4. **Single point NEVPT2 and TDDFT/CAM-B3LYP computation of one sample excited state MD trajectory.** The TDDFT/CAM-B3LYP (left) and NEVPT2 (right) energies are computed for a single trajectory for geometries taken every 5 fs apart. The difference of energies with respect to the ground state energy is plotted for states S_i ($i = 1 - 9$) as function of Fe-C distance (Å).

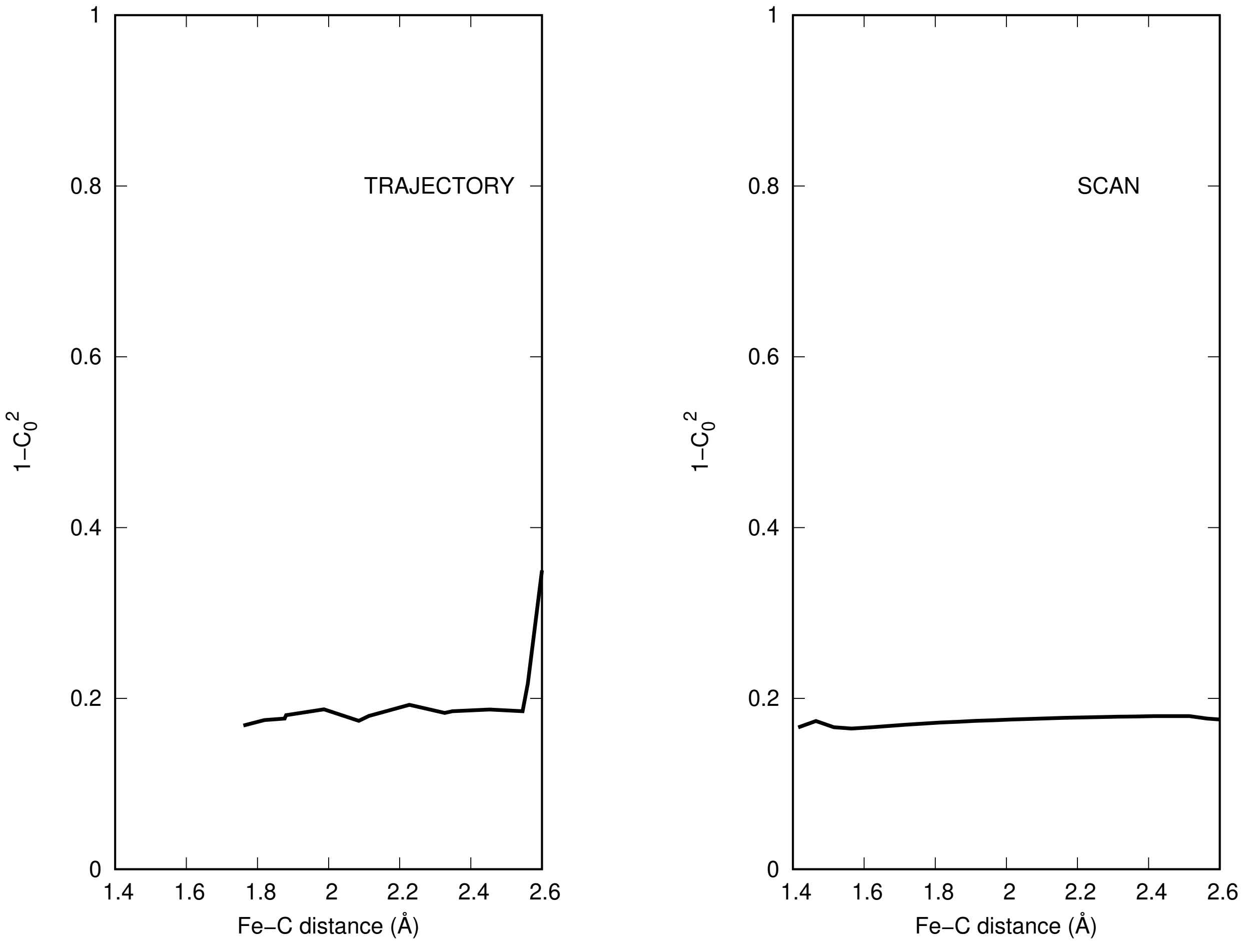

Supplementary Figure 5. **Plot of 1-C_0^2 vs Fe-C distance along axial Fe-CO bond dissociation.** The plot 1-C_0^2 vs Fe-C distance for taken from CASSCF(10e,10o) computation done for geometries from a excited state MD trajectory taken every 5 fs apart (left) and along a Fe-C scan (right) corresponding to Supplementary Figure 3(b).

SUPPLEMENTARY NOTES 2

The problem about break down of TDDFT framework as the S_1 and S_0 potential energy surface approaches each other is very well known. We performed a CASSCF(10e,10o)/NEVPT2 along the Fe-C bond distance and plotted the energies in Supplementary Figure 3(b). Here we present a measure of the multireference character, i.e. plot of (1-C_0^2) vs the Fe-CO bond distance. The C_0^2 is taken as the co-efficient of the closed shell CSF contribution in the ground state wavefunction, i.e. $|2222200000\rangle$. As seen from Supplementary Figure 5 (right panel) the development of multi-reference character in rather limited as the bond elongates and TDDFT can be applicable as far as single/multideterminant description is concerned. However, since the scans are rigid, we have conducted the same computation along one of the trajectories as mentioned earlier when we compare energetics, and plotted (1-C_0^2) vs Fe-C distance in Supplementary Figure 5 (left panel). We clearly see that even though the (1-C_0^2) remain nearly constant and below 0.2 mark most of the region and when Fe-C distance only goes beyond 2.55 Å the system becomes multi-reference. This is also evident from the energies plot with respect to the distance where we see that S_1 and S_0 cross at a distance larger than 2.5 Å. Thus we see that $Fe(CO)_5$ can be described as a single reference system and $Fe(CO)_4$ cannot. Our study focuses on dissociation of $Fe(CO)_5$ to $Fe(CO)_4$ and based on Supplementary Figure 5 we can safely describe the region in between. Based on this limitation of TDDFT we have refrained ourselves of involving S_1 or S_0 dynamics, as based on this diagnostic we see that any description of S_1 state is flawed as S_0 and S_1 energies become quasi-degenerate once the bond breaks. One serious limitation of this piece of work is that we cannot describe the dynamics of the S_1/S_0 state of $Fe(CO)_4$.

SUPPLEMENTARY NOTES 3

Exclusion of Triplets from the ESMD Simulations: The exclusion of triplets from the excited state MD simulation has been motivated primarily from overwhelming evidence from multiple experiments. Three independent articles, based on three different spectroscopic techniques, have ruled out the presence of inter-system crossing (ISC) and triplet pathway in the photodissociation of $Fe(CO)_5$ in the gas phase. Trushin and co-workers [8] have shown in their work that singlet pathway to be operative with no ISC. They have, based on computation of time-constants for the dissociation process, excluded the possibility of ISC happening, arguing that the photodissociation happens much faster than the ISC. Additionally, Wernet and co-workers [10] have corroborated the above claim based on the high-temporal resolution gas phase TR-XPS studies. XPS is very sensitive to the electronic structure of the species, not least to the multiplicity due to strong spin-orbit coupling in the final states of the XPS probe, and would in principle have picked up the formation of triplet species or if the triplet pathway had a major involvement. They have confirmed the absence of any triplet involvement up to 6ps which is beyond the temporal regime of our present studies. Recently using time resolved IR experiments Ramasesha and co-workers have shown that the ISC channels are only operative in the $Fe(CO)_4$ moiety after 15 ps timescale [11]. Thus, gathering from three independent experimental studies that triplet pathways are not operative it is pragmatic for us to exclude the triplet states in the study, and investigation of possible involvement of triplet states for less probable/minor pathways will have to await further investigations and improvements in simulations capabilities.

Also, our study inherently deals with the photo-dissociation, we are already running the risk of breakdown of DFT/TDDFT as the Fe-CO bond dissociates, which we have tried to state clearly in our work. As we know that $Fe(CO)_4$ has a triplet ground state, the inclusion of triplets would furthermore amplify the problem that the DFT/TDDFT already has in dealing with bond dissociation.

Ultrafast ISC has been simulated for other 3d transition metal (TM) complexes which deal with well-known iron based photo-sensitizers and hence have experimentally well-established evidence for the role of triplet states [35, 36]. However, for the system we deal with here ample experimental evidence as discussed earlier tells us not to primarily consider it here for the dissociation dynamics. Nevertheless, in an attempt to find a justification for the singlet pathway and the absence of triplet states in the excited state dynamics following photo-excitation of iron pentacarbonyl in gas phase, we have made Fe-CO scans for both singlets and triplets involved at the NEVPT2 level of theory which is robust in its treatment of triplet and computation of SOC elements. The triplet manifold is an analogue of the singlet manifold, i.e., it comprises of four ^{3}MC states which are dissociative in nature and followed by these are the ^{3}MLCT bound states. In the figure below we show the NEVPT2 PES of the triplet (black lines) and singlet (blue lines). The systems where fast sub 100fs ISC happens, the ISC process happens between bound states having similar shapes (non-dissociative and hence described by LVC model) shown in Ref. [35, 36]). The crossing between the ^{3}MLCT (bound) and ^{1}MLCT (bound) states (denoted by green circle in Supplementary Figure 6) are energetically much higher as compared to the crossing between ^{1}MLCT (bound) states and the ^{1}MC (dissociative) states. Thus the rate for transition from the ^{1}MLCT (bound) states to the ^{1}MC (dissociative) state will be much faster as compared to the ISC rate for the present system. The black circle in the left side figure denotes the crossing between the ^{1}MC (dissociative) state and the ^{3}MLCT (bound) states. The ^{3}MC states which are also dissociative in nature are well separated from the singlet manifold. Now the process involves excitation to a MLCT state followed by decay to dissociative ^{1}MC states followed by fast dissociation, as depicted by the curved arrow in Supplementary Figure 6. The internal conversion within the singlet manifold to the dissociative ^{1}MC state happens before the region where ISC could occur and by the time the system encounters the ISC region (highlighted by the black circle), the system is already in the process of a fast dissociation. Thus, the system likely spends too little time in that region for ISC to effectuate, or in other words the system has too large a nuclear velocity, which is an important factor in ISC rate, for ISC pathway to be competitive. Though the spin-orbit coupling (SOC) between a ^{1}MC and ^{3}MLCT states is expected to be low, when computed we found it to the order of 220 cm^{-1}, at NEVPT2 level of theory. Considering the timescale associated to the SOC is $\sim$75 fs under ideal conditions, and given the system only spends much less time in the resonant region due to the fact that singlet states are dissociative and triplet states are bound, we would assume that the branching ratio to the triplet state is rather low. This provides a justification for the experimentally observed singlet pathway. Alternatively nuclear wavepacket overlap, which is an important aspect in the ISC process, can also assumed to be low given the different shapes of the crossing dissociative singlet ^{1}MC PES and bound triplet ^{3}MLCT PES. This is again in stark difference to the two cases where fast ISC happens [35, 36]. The scan at the TDDFT/CAM-B3LYP level in Supplementary Figure 6 also gives the same overall behavior for the singlet and triplet manifolds although the ^{3}MLCT states are less dispersed than the ^{1}MLCT states. Notice also that the crossings between the ^{1}MC and ^{3}MLCT states occur in a region where the dissociation has already been initiated.

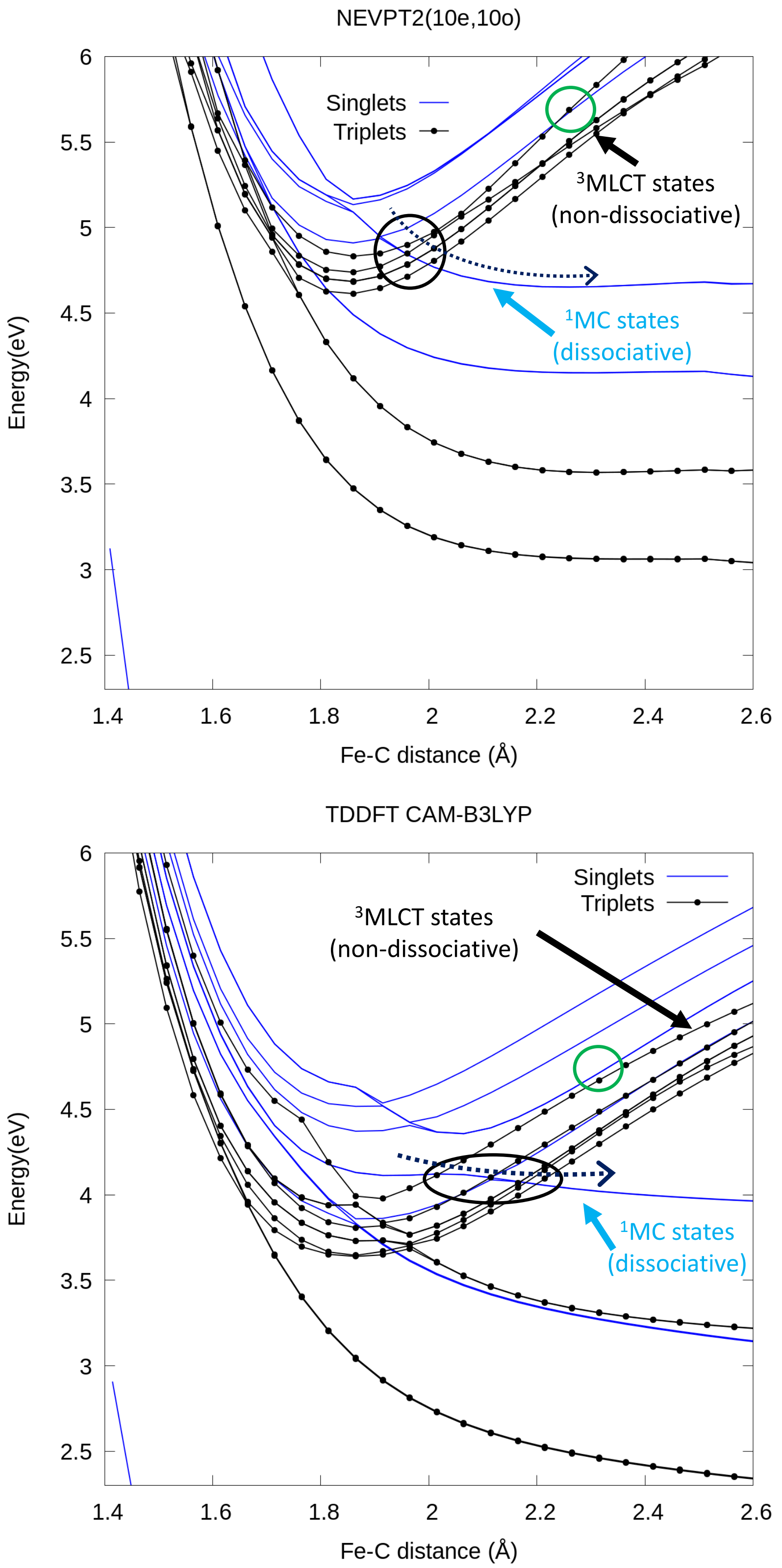

Supplementary Figure 6. **Adiabatic potential energy surfaces of singlet and triplet manifold along a rigid scan corresponding to the dissociation of axial Fe-CO bond.** (a) Top panel: NEVPT2 adiabatic potential energy surfaces of the 9 lowest lying electronic singlet (blue) states and 9 lowest triplet (black) states plotted against varying an axial Fe-C distance starting from the equilibrium D_{3h} geometry (see Methods Section), The black circle denotes the crossing region between the singlet ^{1}MC state and triplet ^{3}MLCT states. The green circle denotes the crossing region between ^{1}MLCT states and ^{3}MLCT states. The curved arrow signifies the path that dissociation happens in the ^{1}MC state. (b) Bottom panel: TDDFT/CAM-B3LYP adiabatic potential energy surfaces for the lowest singlet (blue) and triplet excited (black) states.

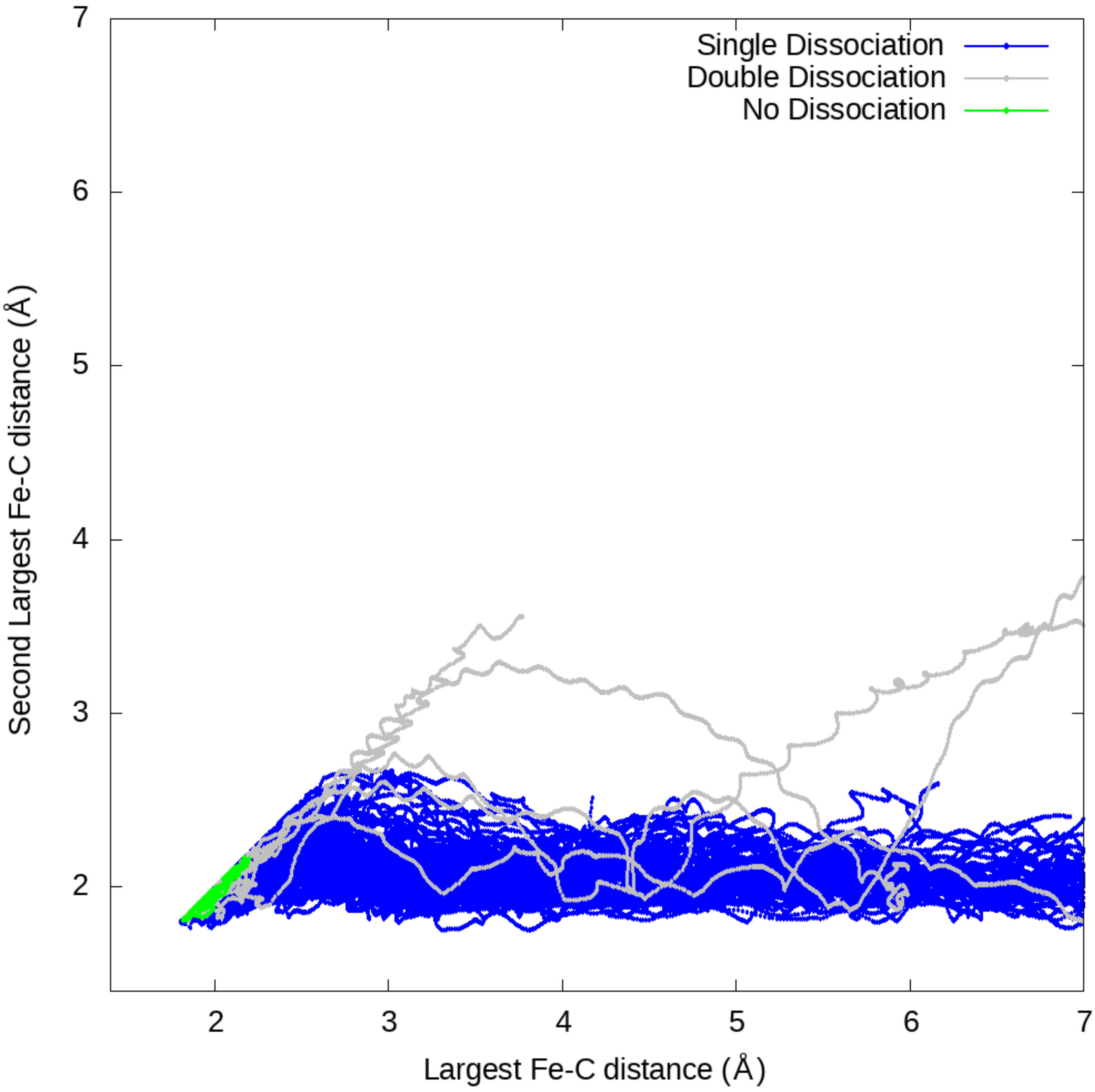

Supplementary Figure 7. **A two dimensional scatter plot of the two largest Fe-C distances during the ESMD in the 116 trajectories initiated from the S_6 state.** Blue lines depict trajectories which show release of a single CO group, whereas green lines indicate no dissociation. The gray lines represent trajectories showing double Fe-C bond dissociation, or simply deviates strongly from other single dissociation trajectories.

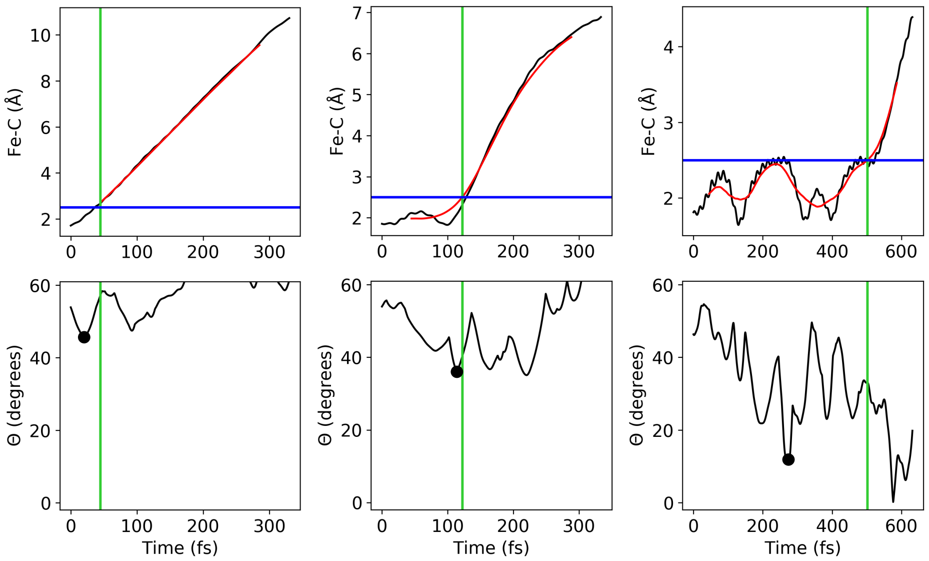

Supplementary Figure 8. **Traces of the Fe-C distance of the released CO and the pseudorotation distortion angle (Θ) as seen in three example trajectories from the 116 ESMD trajectories initiated from the S_6 state.** In red a running average over a ±45 fs interval of the Fe-C distance is presented. The running average partially quenches rapid distance variation, which allows us in every trajectory to identify a unique time of dissociation $\tau_{\rm dissoc}$, when the Fe-C distance elongates beyond 2.5 Å. (The interval for averaging is motivated by the 90 fs oscillation period observed in Figure 2 and Supplementary Figure 9, and 2.5 Å is the lowest distance giving a unique time of dissociation for all trajectories (i.e. in which the red line only crosses the blue threshold line once.) This time of dissociation $\tau_{\rm dissoc}$ (highlighted by a vertical green line) is used in Figure 2 for measuring the release rate and in Figure 5 to track the smallest value (highlighted with a red dot) of the pseudorotation distortion angle Θ within [0,$\tau_{\rm dissoc}$].

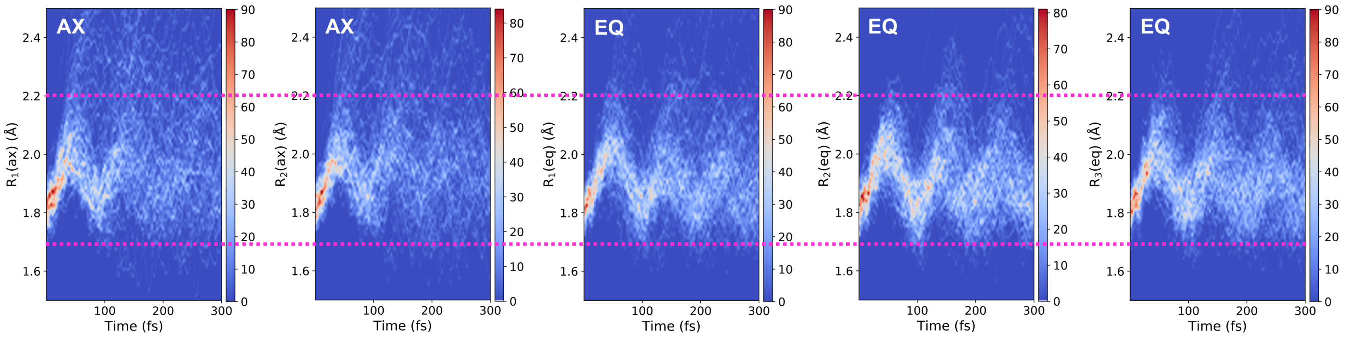

Supplementary Figure 9. **Variation of Fe-C distance for $Fe(CO)_5$ as function of time.** Individual distributions of the five axial and equatorial Fe-C_i distances as a function of time for all 110 trajectories. The scale on the heat map is the number density (1/Å).

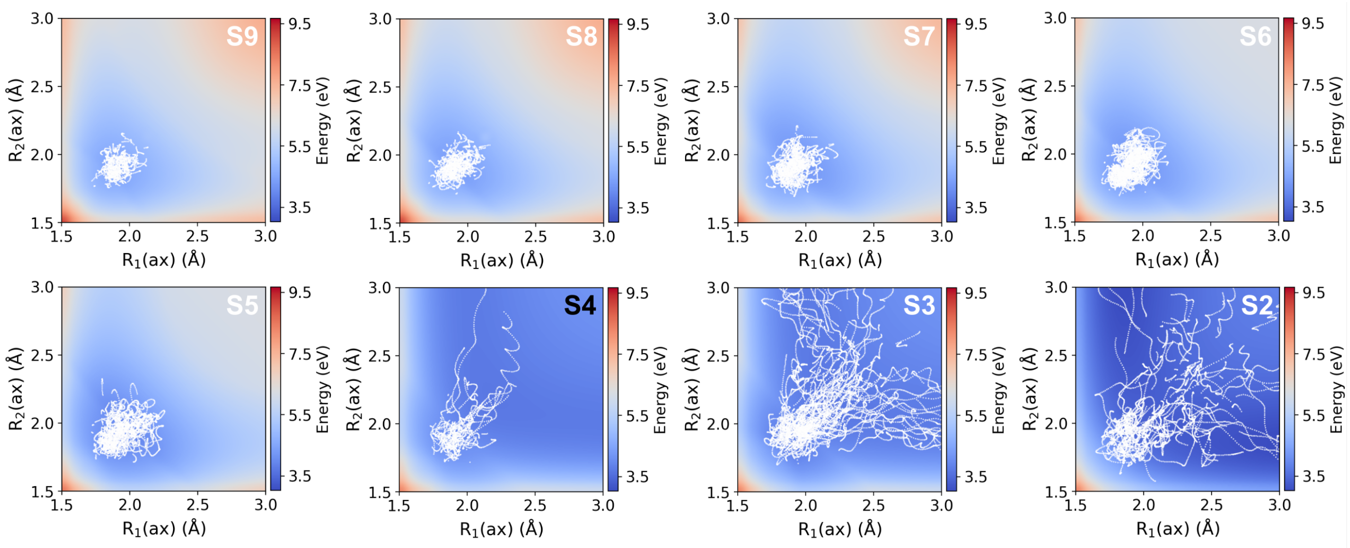

Supplementary Figure 10. **Superposition of distribution of axial Fe-C R_1(ax) vs R_2(ax) distances over the two dimensional cuts in the potential energy surfaces.** Two dimensional cuts in the potential energy surfaces of S_i for $i = 2 - 9$ obtained by TDDFT scans along the axial Fe-C R_1(ax) vs R_2(ax) distances are shown here. These two dimensional cuts correspond to the one dimensional cuts presented in panel a in Supplementary Figure 3. In white lines, scatter plots of the 110 single dissociative trajectories are superimposed. S_4 forms a special case where we actually show only the part of the trajectory prior to transitions on to the S_3 state, and excluding back transfer from S_3. This is done to highlight that most dissociation happen on S_3 onward, though S_4 is dissociative in nature.

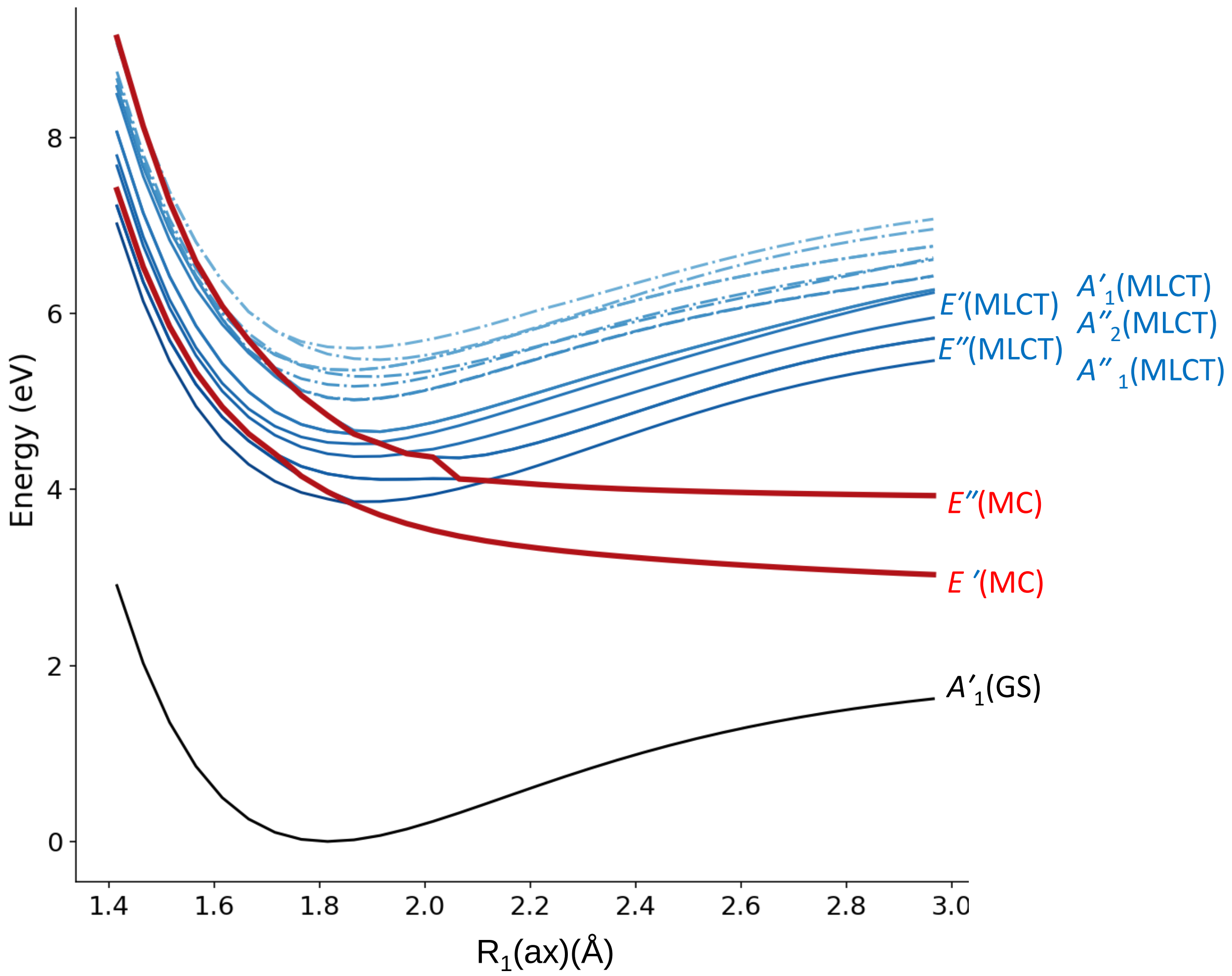

Supplementary Figure 11. **Diabatic potential energy surfaces for rigid scan corresponding to the dissociation of axial Fe-CO bond.** Approximate diabatic TDDFT potential energy surfaces of the 10 singlet states plotted against varying an axial Fe-C distance starting from the ground state D_{3h} geometry. Assigned to MLCT and MC character respectively, the dissociative diabatic states are shown in red and the non-dissociative diabatic states are shown in blue. Based on the data of the adiabatic energies from panel a in Supplementary Figure 3.

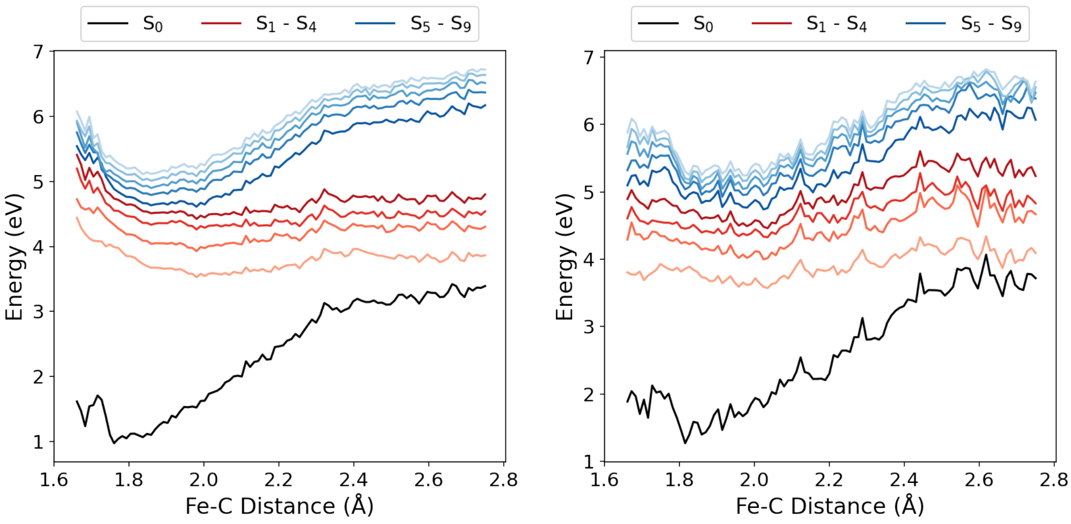

Supplementary Figure 12. **Dynamically averaged adiabatic potential energy surfaces along Fe-CO bond dissociation of sampled geometries from excited state molecular dynamics(ESMD) trajectories.** The potential energy of each the states along the ESMD simulations was sampled as a function of Fe-C distance,corresponding to the released CO, and averaged separately for the 94 axial (left) and 16 equatorial (right) dissociative trajectories. At long Fe-C distance, non-dissociative (MLCT) and dissociative (MC) states are colored blue and red, respectively. The PES averaged over all 110 singly dissociative trajectories are given in Figure 4(a) in the main manuscript.

SUPPLEMENTARY NOTES 4

POPULATION DYNAMICS AND NON-ADIABATIC DYNAMICS: The time spent in a state averaged over the trajectories can be thought of as a measure of the lifetime of the state and they are listed in Supplementary Table 2. Furthermore, looking at the number of jumps in the surface hopping matrix in Supplementary Table 3, we notice that hops predominantly occur to adjacent adiabatic states i.e. from S_n to $S_{n\pm1}$, even though the surface-hopping algorithm is not restricted to these transitions. Based on this we did kinetic modeling (see the schematic Supplementary Figure 14) for the population dynamics. It is noticeable here from Supplementary Tables 2 and 3 that we also take into account that the system can hop up from S_6 state to S_{7-9} states, which can be due to the fact that states can relax along different degrees of freedom and become lower in energy than the initial energy of the system. The fitted curves, of electronic population are overlayed with the simulated data as shown in Supplementary Figure 13. The area under fitted curves were taken as a measure of lifetime of different states (since due to the presence of back reactions exponential fits were not an option) and was found to match well with the lifetime data from the simulation (see Supplementary Table 2). This also establishes that the dynamics can be accurately described in a simple model, in which hopping from one state predominantly happens to the adjacent adiabatic states.

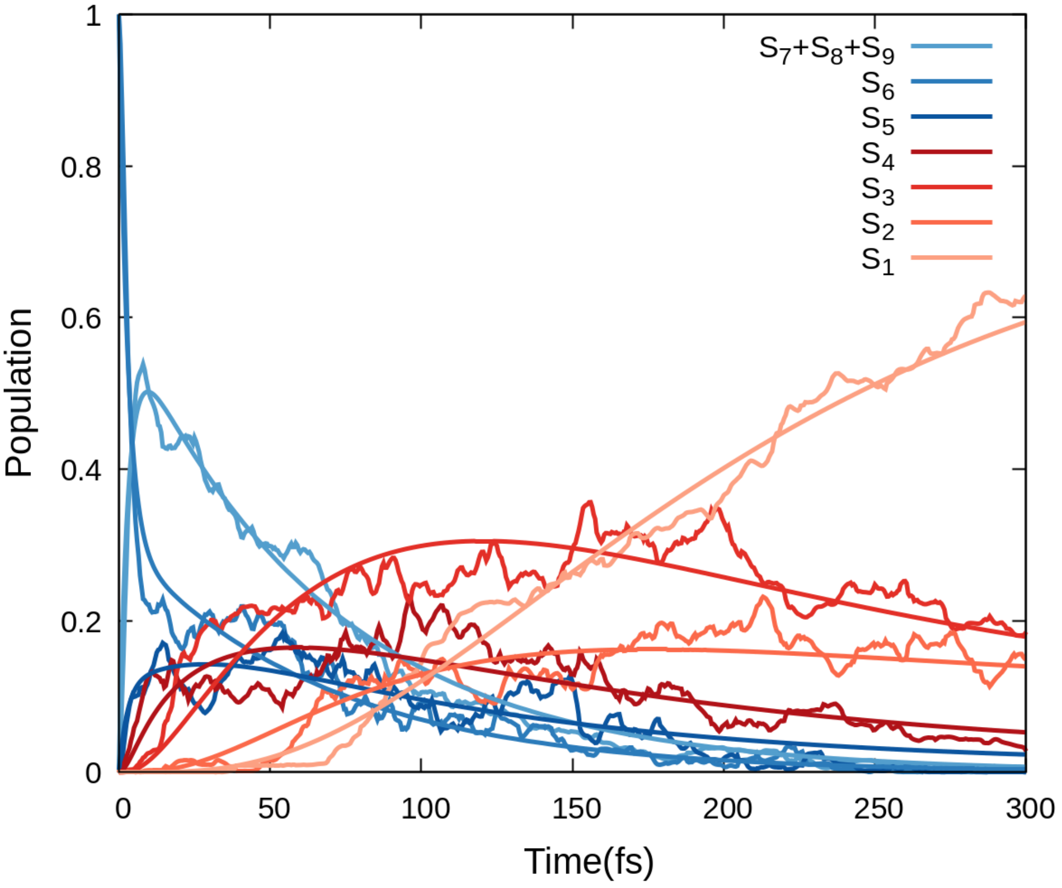

Supplementary Figure 13. **Excited state population dynamics.** The population dynamics, for 110 single dissociation trajectories, of the adiabatic electronic states are shown along with the smooth curves obtained by fitting the simulation data with the kinetic model as shown in Supplementary Figure 14. The dissociative and non-dissociative states are shown with shades of red and blue respectively which matches with the plot of potential energy surfaces in Supplementary Figure 3.

State	Lifetime (fs) from simulation	Lifetime (fs) from kinetic model
S_{789}	34	39
S_6	22	23
S_5	18	22
S_4	31	32
S_3	77	68
S_2	47	36

Supplementary Table 2. **Table showing the lifetime of different adiabatic states involved in the simulation and kinetic modeling.** The dissociative and non-dissociative states are shown with red and blue respectively. In our short simulations, the system eventually goes to the S_1 state and stays there, hence the lifetime for S_1 does not have a meaning.

State	S_2	S_3	S_4	S_5	S_6	S_7	S_8	S_9
S_1	**146**	7	0	0	0	0	0	0
S_2	–	**183**	6	2	0	0	1	1
S_3	**87**	–	**184**	28	10	2	0	1
S_4	4	**104**	–	**149**	21	8	3	2
S_5	0	10	**82**	–	**154**	31	4	1
S_6	2	1	10	**81**	–	**170**	23	14
S_7	0	1	4	12	**171**	–	**149**	24
S_8	1	0	1	6	41	**124**	–	**98**
S_9	0	0	0	4	20	26	**91**	–

Supplementary Table 3. **Table for the hopping matrix for the states involved in the ESMD simulations.** Bold numbers indicate the number of hoppings to the next neighbor state, i.e. from S_n to $S_{n\pm1}$.

SUPPLEMENTARY NOTES 5

The kinetic model was set up as a series of rate equations of the form

$$\frac{dN_j}{dt} = k_{ij}N_i + k_{kj}N_k - (k_{ji} + k_{jk})N_j, \tag{1}$$

where $i+1 = j = k-1$, $2 \leq j \leq 6$, and N_j is the population of the state j. For the special cases of $j = 1$ and $j = 7, 8, 9$, the equations reduce to

$$\frac{dN_1}{dt} = k_{21}N_2 - k_{12}N_1 \tag{2}$$

and

$$\frac{dN_7}{dt} = k_{67}N_6 - k_{76}N_7, \tag{3}$$

respectively (the 7, 8, 9 states are considered together). The initial population $N_j(t), t \leq 0$ was set to 0 for all states except $j = 6$.

The rate equations were numerically integrated using the `solve_ivp` ODE solver of Scipy 1.5.0 [38] and a 4-5th order Runge-Kutta method [39]. The resulting time-dependent populations were fitted to the data using Scipy's least-squares fitting routine using the `trf` (trust-region reflective) algorithm [40].

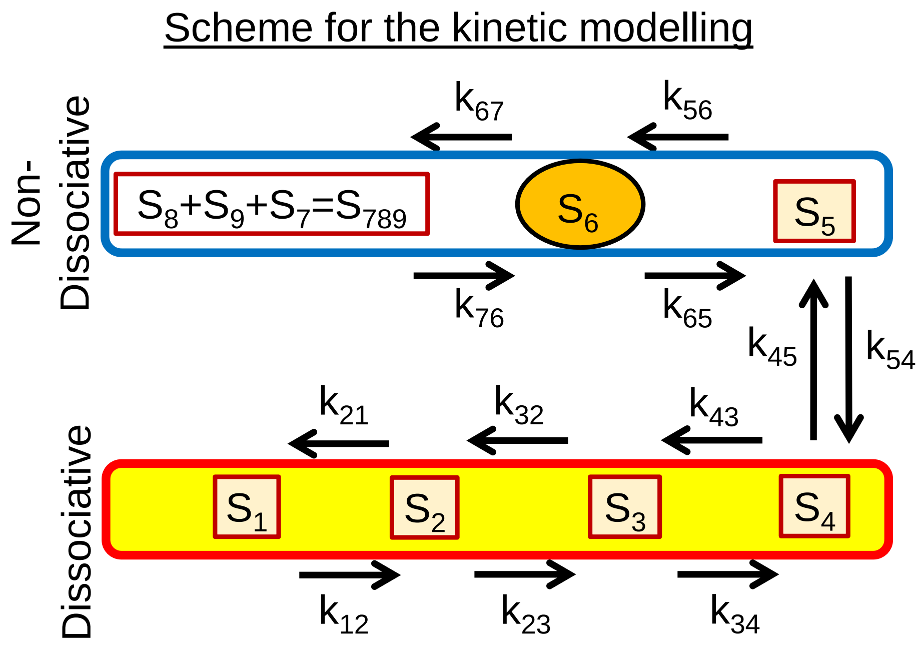

Supplementary Figure 14. **The scheme employed for the kinetic modeling.** The scheme is based on the hopping matrix data as shown in Supplementary Table 3. The forward and backward rate constant used in the modeling are shown below and above the arrows.

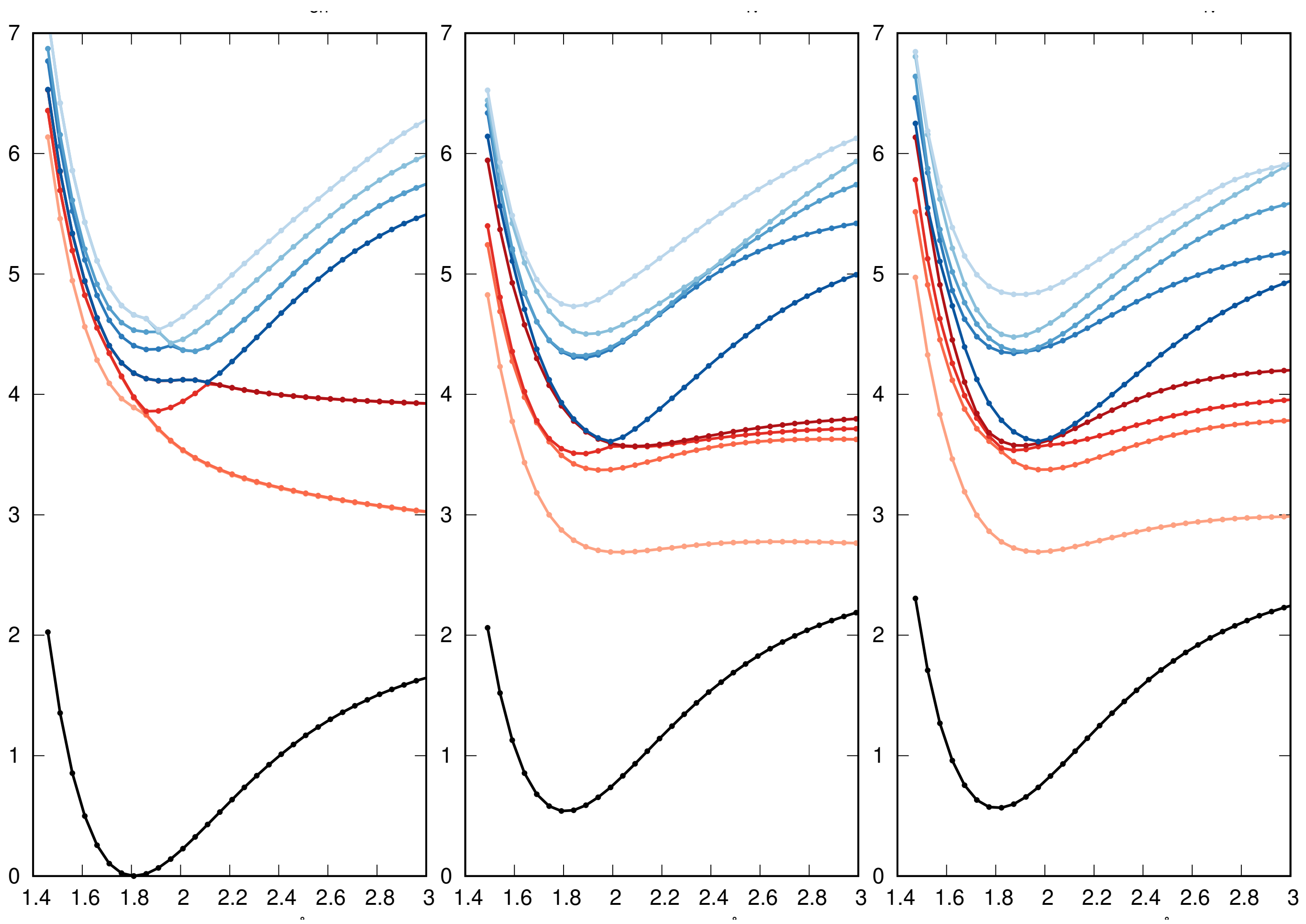

Supplementary Figure 15. **The adiabatic TDDFT potential energy surfaces of all the states plotted against varying Fe-C distance starting from D_{3h} geometry (left side) and near C_{4v} geometry (middle and right).** The middle and right panel represent the dissociation of CO which was axial and equatorial respectively in the D_{3h} geometry and distorted (angular) to near C_{4v} geometry. The dissociative and non-dissociative states are shown with shades of red and blue respectively which matches with the plot of the potential energy surfaces in Supplementary Figure 3.

	S_0 (S_6 at S_0 geometry) CAM-B3LYP/def2-TZVP//CASPT2(12,12)/TZVP			S_5 CAM-B3LYP/def2-TZVP//CAM-B3LYP/def2-TZVP		
bond	freq (cm^{-1})	intensity (km/mol)	character	freq (cm^{-1})	intensity (km/mol)	character
Fe-C	419(**443**)	0	A_1' sym stretch ax+eq	218	4	$\sim B_1$ sym stretch eq
Fe-C	446(447)	0	A_1' sym stretch ax-eq	312	6	$\sim E$ stretch asym eq
Fe-C	498(450)	30(5)	E' asym stretch eq	**363**	13	$\sim A_1$ sym stretch eq
Fe-C	498(450)	30(5)	E' asym stretch eq	**477**	27	$\sim A_1$ stretch axial
Fe-C	500(465)	29(142)	A_2'' asym stretch ax	784	31	$\sim E$ asym stretch eq
C-O	1971(2077)	1349(182)	E' asym stretch eq	2101	2295	$\sim E$ asym stretch eq
C-O	1971(2077)	1349(182)	E' asym stretch eq	2108	484	$\sim B_1$ sym stretch eq
C-O	2000(1951)	1511(4146)	A_2'' asym stretch ax	2130	1587	$\sim E$ asym stretch eq
C-O	2000(1999)	0	A_1' sym stretch ax-eq	2157	678	$\sim A_1$ stretch ax
C-O	2081(2101)	0	A_1' sym stretch ax+eq	2928	288	$\sim A_1$ sym stretch eq

Supplementary Table 4. **Table for the relevant frequency corresponding to the Fe-C vibration and C-O vibration in optimized geometry S_0 (D_{3h}) and S_5 (approximately C_{4v}).** Symmetry classification of the vibrations is used in S_0 (D_{3h}), and used to indicate the motion in the vibrational modes in S_5 even though the E double degeneracy is completely lifted. The symmetric Fe-C stretching modes in the S_5 state are highlight in bold.

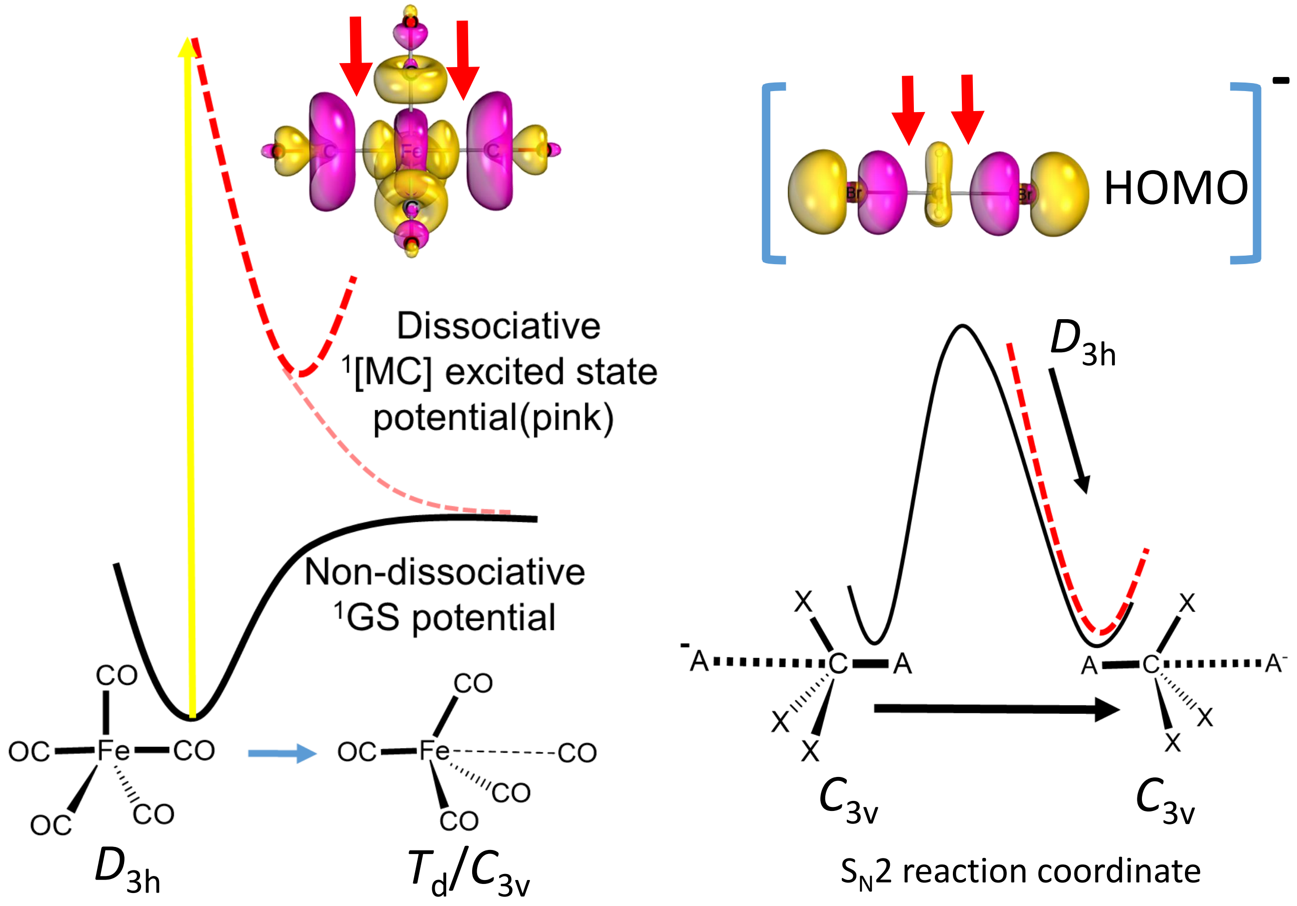

Supplementary Figure 16. **The correspondence between the potential energy surface of a S_N2 reaction and that of photodissociation of the $Fe(CO)_5$.** The right hand panel shows the well known energy profile of the S_N2 reaction and the orbital overlap symmetry of the frontier molecular orbital. The left hand figure shown the similarity from symmetry and overlap symmetry point of view for $Fe(CO)_5$. The highest occupied molecular orbital (HOMO - doubly filled) for S_N2 transition state has been shown here. This orbital has the same anti-bonding overlap symmetry that we see in the metal centered $3d_{z^2}$ orbital shown in the left, which is singly occupied in the dissociative MC excited states.

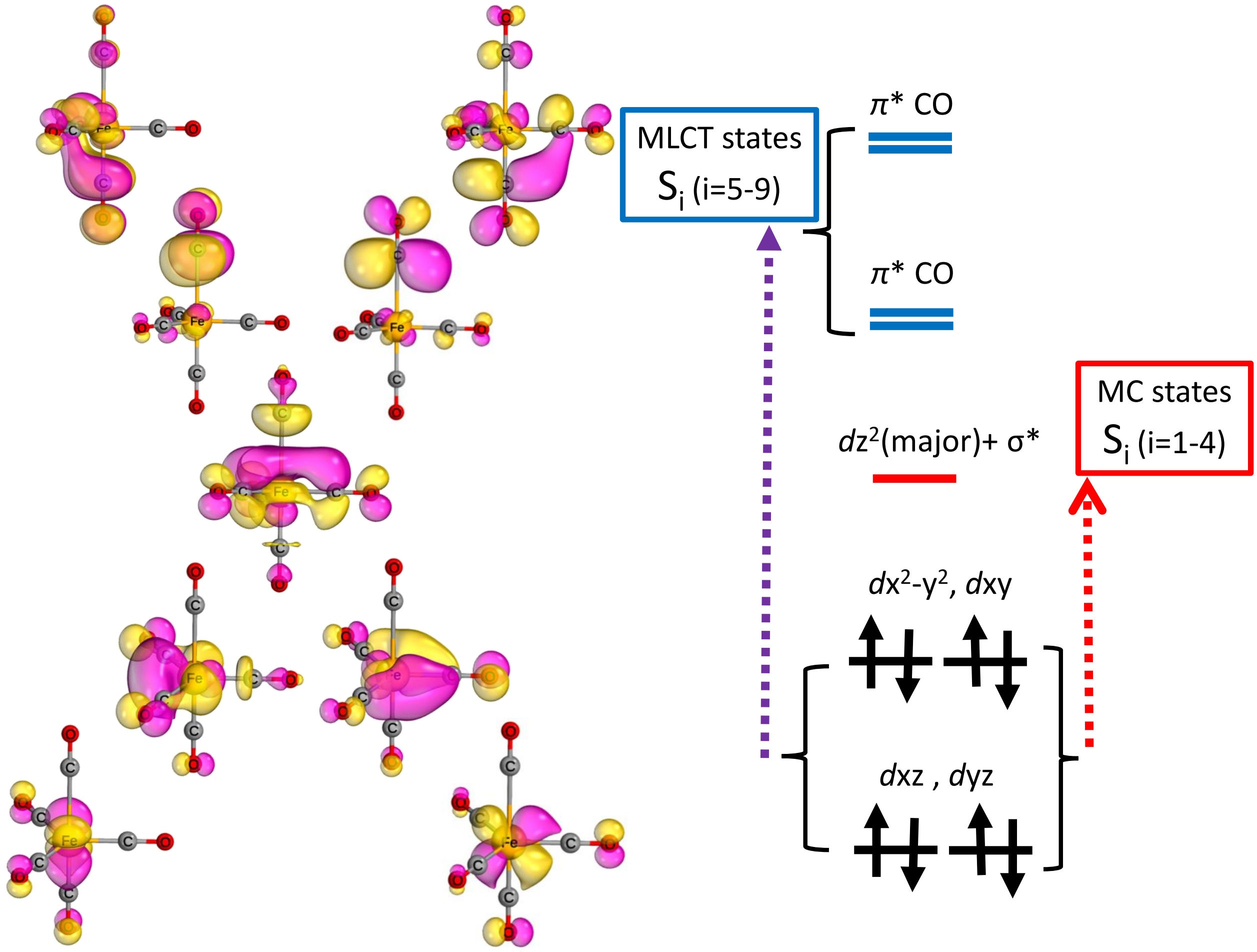

Supplementary Figure 17. **Frontier molecular orbitals involved in the photodissociation of ironpentacarbonyl at a geometry with one increased axial Fe-C distance (2.5 Å), relative to the ground state geometry.** Relevant connection between the electronic states and subsequent dissociation dynamics are summarized in the right part of this figure. The figure serves the purpose of connection between the orbital (one electron) picture with the electronic state (many electron) picture. As depicted here in the orbital picture, and in Supplementary Figure 11 in a state picture, as the Fe-C(axial) bond is stretched the lowest four (dissociative) states possess the MC character where as the higher lying bound states have MLCT character.
